# Comparison of Open Repair and Laparoscopic Percutaneous Internal Ring Suturing Method in Repairing Inguinal Hernia in Children

**DOI:** 10.7759/cureus.14262

**Published:** 2021-04-02

**Authors:** Yusuf A Kara, Beytullah Yağız, Özlem Balcı, Ayşe Karaman, İsmet F Özgüner, İbrahim Karaman

**Affiliations:** 1 Pediatric Surgery, Dr. Sami Ulus Health Research and Training Center, Ankara, TUR

**Keywords:** inguinal hernia, herniorrhaphy, children, laparoscopy

## Abstract

Introduction

An inguinal indirect hernia is one of the most frequent surgical conditions in children. In this study the experience with laparoscopic percutaneous internal ring suturing (PIRS) and open inguinal hernia surgery and their relations evaluated.

Methods

All children over 90 days of age and without having prior inguinal region surgery with a diagnosis of indirect inguinal hernia underwent surgical repair with open or laparoscopic PIRS technique. Patients' gender, age at surgery, inguinal hernias side, surgery duration, recurrence, complications, and follow-ups were collected.

Results

A total of 488 inguinal hernias of 405 patients were repaired. The diagnoses were unilateral inguinal hernia in 360 (88.9%) and it was bilateral in 33 (8.1%) patients. The operative technique was laparoscopic PIRS for 227 and open inguinal hernia surgery for 178 patients. In the PIRS group, a contralateral hernia was found in 48 of 205 children (23.4%). The surgery times were 23.3 (PIRS) and 33.7 (open) min for unilateral and 28 (PIRS) and 53.1 (open) min on average for bilateral inguinal hernia surgery. Mean follow-up was 30.4 months for PIRS and 24.4 months for open technique. Recurrence was observed in seven (3%) patients in PIRS and one (0.5%) in the open group and postoperative complications in three (1.3%) in PIRS and four (2.2%) in the open group.

Conclusion

PIRS method has the advantage to evaluate contralateral hernia at the same session, minimal scar related to surgery, and preserve the spermatic cord from manipulation. PIRS is an alternative feasible method and easy to perform to repair the inguinal hernia with/without communicating hydrocele in children.

## Introduction

In childhood, one of the most frequent surgical pathologies is indirect inguinal hernia [1]. For centuries, the surgical technique to correct this anomaly has been open inguinal hernia repair [2]. In the late 1990s, the first laparoscopic repair of adult inguinal hernia was reported [3]. Subsequently, different approaches for laparoscopic inguinal hernia repair have been described [4-6]. In adults, three-port laparoscopic methods were reported first, and thereafter, percutaneous internal ring suturing (PIRS) via a single port was introduced in the pediatric population [7]. The advantages of this method are the chance to evaluate contralateral inguinal hernia, better cosmetic outcomes, and performing the procedure without manipulating the spermatic cord and testicular vessels. However, complications such as recurrence and postoperative hydrocele were claimed to be higher in children who underwent the PIRS procedure than in those who underwent open surgery [8]. The primary aim of this study is to compare recurrence and postoperative complication rates of the PIRS technique with those from open surgery. Outcomes such as surgical time, anesthesia time, and contralateral incidental inguinal hernia presentations were also evaluated. 

## Materials and methods

The study was performed in adherence to the current form of the Declaration of Helsinki. Ethical committee approval was granted by Health Sciences University Dr. Sami Ulus Ethical Committee, with the IRB number of 2020/01-2. Between February 2014 and November 2019, a total of 479 children underwent surgery for inguinal hernia. The operative technique was laparoscopic PIRS for 301 patients (74 patients excluded; 14 of due to prior inguinal surgery, 60 of due to under 90 days of age), and open inguinal hernia surgery was performed on 178. When the patients presented to the hospital with an inguinal hernia, the parents were informed about the laparoscopic and open repair procedures. Open surgical technique and laparoscopic technique were fully explained, and the choice of method was made by parents. In the laparoscopic group, 14 patients who had prior inguinal surgery and 60 patients who were under 90 days of age were excluded from this study due to being subjects in another study. In this study, we evaluated a total of 405 patients - 227 who had laparoscopic PIRS and 178 who had open inguinal hernia repair. In all laparoscopic procedures, the method of choice was the PIRS method described by Patkowski et al. We collected the patients’ data retrospectively. All data were analyzed using IBM Statistical Package for the Social Sciences Statistics (SPSS) for Windows version 20 (IBM Corp., USA).

Surgical technique

Laparoscopic percutaneous internal ring suturing method and open surgical method (Bassini procedure) is preferred to repair the inguinal hernia for all patients.

Laparoscopic PIRS Method

Patients were placed in supine position under general anesthesia. After the application of local anesthesia with lidocaine, an umbilical vertical incision was made. A 5 mm umbilical trocar was placed via the open technique. Intraabdominal pressure was set between six and 12 mm CO_2_ according to the child’s weight and age. Laparoscopic exploration was made via a 5 mm telescope with a 30° angle. After internal inguinal ring exploration, a 2 mm incision was made to the skin at the inguinal ring level. A 20-G spinal needle or 20-G to 22-G angiocath needle was used to access the preperitoneal cavity. A 2/0 or 3/0 nonabsorbable braided suture was prepared on the needle for ligation. A needle with a suture was inserted through the incision and reached the preperitoneal cavity. First, the suture curved from the medial side of the internal inguinal ring, and then, the second suture curved from the lateral side. The peritoneum over the spermatic cord and vessels was dissected with caution. Two loops were seen in the abdominal cavity, and one of the two was passed through another loop and taken out of the abdominal cavity. The sutures were tied outside and the skin incision sutured with 5/0 absorbable sutures. The closure of the internal inguinal was checked laparoscopically. The fascia of the umbilical incision was closed using 2/0 or 3/0 absorbable sutures, and the skin incision was sutured with 5/0 absorbable sutures. Enteral feeding started on the second postoperative hour, and patients were discharged from the sixth to the eighth postoperative hour. Patients’ data were collected and recorded.

Open Technique

Patients were placed in a supine position under general anesthesia. The external ring was revealed by dissecting medially along the Poupart’s ligament via an inguinal transverse incision. The hernia sac was located in the anteromedial region of the testicular cord. Cord, testicular vessels, and vas deferens were separated using fine-tissue forceps, and the hernia sac was clamped and dissected up to the preperitoneal fat tissue. After confirmation that there were no sliding organs, the hernia sac was twisted and double-suture ligated with 2/0 or 3/0 absorbable sutures. Fascia and layers were closed in order with absorbable sutures.

## Results

A total of 405 patients included in this study. The operative technique for 227 of these patients was laparoscopic PIRS and open surgical method was chosen for 178 of all.

Laparoscopic PIRS method

Two hundred twenty-seven patients with 299 inguinal hernias underwent the PIRS procedure; 144 were boys (63.4%) and 83 were girls (36.6%). The mean age at surgical intervention was 4.56 ± 3.74 years (91 days to 17 years). At the time of presentation to the hospital, 215 patients' (94.8%) physical examinations were concordant with an inguinal hernia with/without communicating hydrocele. The remaining 12 (5.2%) were due to other reasons besides inguinal hernias and were diagnosed and repaired intraoperatively. Preoperative examination showed inguinal hernia on the right side in 129 patients (56.8%), the left side in 67 patients (29.5%), and in 19 patients (8.3%), the findings were indicative of bilateral inguinal hernia. In the incidentally determined group, after intraoperative approval of the family, the inguinal hernias of these patients were repaired. Five (2.2%) had a right-sided inguinal hernia, two (0.8%) had a left-sided inguinal hernia, and five (2.2%) had bilateral inguinal hernias. Other than these, 48 patients (21.1%) were intraoperatively diagnosed with a contralateral inguinal hernia without having preoperative symptoms - 25 on the right side (52%) and 23 on the left side (48%). All procedures were completed in one session and laparoscopically. The average surgery time was 23.4 ± 5.31 min for unilateral and 26.5 ± 7.11 min for bilateral inguinal hernia repair. The mean follow-up period was 30.4 months (six to 69 months). Recurrence was seen in seven patients (3%), three were repaired with open surgery due to the patient’s parents’ concerns about laparoscopy, and four were repaired with the PIRS method again. No recurrence was seen in these reoperations. Intraoperative hematoma due to puncture of testicular vessels was observed in six patients (2.6%). Bleeding was limited with extracorporeal blunt compression. Three patients (1.3%) developed stitch abscesses cured with drainage and oral antibiotic therapy (Table 1).

Open surgical method

One hundred seventy-eight patients with 189 inguinal hernias were operated on with the open surgical technique; 123 were boys (69.1%) and 55 were girls (30.9%). The mean age at surgical intervention was 4.19 ± 3.34 years (91 days to 14.5 years). The patients were intervened for right-sided inguinal hernias in 104 cases (58.4%), for left-sided inguinal hernias in 63 cases (35.4%), and 11 patients (6.2%) were operated for bilateral inguinal hernias. The average surgery time was 33.6 ± 11.1 min for unilateral and 53.1 ± 12.4 min for bilateral inguinal hernia repair. The mean follow-up period was 24.4 months (six to 68 months). Recurrence was seen in one patient (0.5%) and repaired with the open technique; no intraoperative complication was observed in this group. In the postoperative period, four patients (2.2%) had complications-two (1.1%) developed incisional abscesses and were cured with drainage and oral antibiotic therapy, and wound dehiscence was seen in two patients (1.1%), repaired with primary suture (Table 1).

**Table 1:** Comparison of laparoscopic PIRS method and open surgery for pediatric inguinal hernia repair. PIRS: percutaneous internal ring suturing

	Laparoscopic PIRS		Open surgery	p-Value
N	227		178	
Age (Years)	4.56 ± 3.74		4.19 ± 3.34	0.409
Gender (Male/Female)	144/83		123/55	0.233
Side	Preoperative	Postoperative (Incidental)		
	Right: 129	Right: 109 (5)	Right: 104	
	Left: 67	Left: 46 (2)	Left: 63	
	Bilateral: 19	Bilateral: 72 (5)	Bilateral: 11	
	Negative: 12	N/A	N/A	
Operative Time (min) Unilateral	Mean: 23.4 ± 5.31 (13-38)		Mean: 33.6 ± 11.1 (13-68)	<0.001
Operative Time (min) Bilateral	Mean: 26.5 ± 7.11 (20-65)		Mean: 53.1 ± 12.4 (30-70)	<0.001
Intraoperative Complications	6 (6 hematomas)		0	=0.029
Postoperative Complications	3 (3 stitch abscesses)		4 (2 incisional abscesses, 2 wound dehiscence)	=0.480
Recurrence	7 (3 open repair, 4 re-PIRS)		1 (open repair)	=0.071
Follow-up (Months)	30.4 (6-69)		24.4 (6-68)	<0.001

## Discussion

Open surgical repair has been considered the gold standard for pediatric inguinal hernias and one of the most frequently performed operations in children [1,9]. In recent years, minimally invasive operative techniques have become preferred for inguinal hernia repair in the pediatric population [10]. The open surgical technique provides high ligation of the hernia sac and shows low recurrence, but the procedure involves dissection of vessels, the ilioinguinal nerve, and the spermatic cord, and there is a risk of laceration or injury [11,12]. Although no complications were seen intraoperatively, palpation and pulling up the spermatic cord and vessels could also have a negative effect on productivity and fertility due to microvascular trauma [13].

Recent studies have shown that the recurrence rate varies between 0% and 5% for laparoscopic inguinal hernia repair [14,15]. In the literature, various laparoscopic approaches for inguinal hernia have been reported. The first authors to use laparoscopy for inguinal hernia repair reported a three-port laparoscopic approach [3,16,17]. Following that technique, the subcutaneous endoscopically assisted ligation method was described, which includes a single-port optical view and a suture-holding system [18]. In the PIRS method preferred in this report, the only requirement is a single port for a telescope and a suture with a spinal needle or an angiocath [7]. The complication rate was 2.6% and the recurrence rate was 3% in our study with 227 patients and 299 inguinal hernia repairs using the PIRS method. We used absorbable sutures in seven cases, propylene nonabsorbable sutures in 77 cases, and nonabsorbable braided sutures in 215 cases. Although some reports have shown that the use of absorbable sutures leads to higher recurrence, in our experiment with seven cases, there were no recurrences after a mean 30-month follow-up [19]. This could be the result of a small cohort size.

Detecting metachronous hernias is another advantage of laparoscopic repair. Although contralateral exploration through the hernia sac in open repair is feasible, it may not be possible in all cases. In a large series of open inguinal hernia repairs, contralateral hernias were reported in between 10% and 30% [20,21]. Gollu et al. reported a series with 705 children and stated that their failure rate of contralateral examination was nearly 30% due to various reasons [20]. In 479 children in whom they managed to check the contralateral inguinal region, they found 136 contralateral hernias (28%) [22]. In our study, 48 (24.8%) children in the PIRS group were diagnosed with bilateral inguinal hernia intraoperatively, while their primary diagnosis was a unilateral inguinal hernia. In the open repair group, contralateral exploration was not performed in any child due to lack of experience, and five of 178 patients (2.8%) were operated on for a contralateral inguinal hernia in the postoperative follow-up period. 

The injury of iliac or testicular vessels is a possibility in both the PIRS technique and open inguinal hernia repair technique. In the open surgical technique, it is possible to damage the vas deferens because of its vulnerability; attention must be paid to the incidence of vas injury-the incidence of unilateral vas deferens obstruction was reported to be 26.7% for patients with infertility and a history of open inguinal hernia surgery in childhood [22]. With greater experience, complication rates will decline, and completing the procedure with no complications will be possible. It has been reported in several studies that the more experience a surgeon gains in laparoscopy, the fewer complications, fewer recurrences, and shorter operative times are encountered [23,24]. It has also been reported that the PIRS method has no negative effect on the vascularization of testicles [25,26]. Laparoscopic repair has the advantage of minimizing the postoperative recovery period, which means fewer days unable to work for parents, and children, fewer days out of school [27].

The duration of laparoscopic surgery was 23.3 min for unilateral hernias and 28 min for bilateral inguinal hernias in our study. Some studies have reported 14.3-28 min for unilateral and 20.4-43.6 min for bilateral inguinal hernia repair [7,11,28,29]. The operative times may decline through greater experience with the procedure. In open inguinal hernia surgery in our study, the duration of the operation was 33.7 min for unilateral and 51.3 min for bilateral inguinal hernia repair. In the literature, times are reported as 30.1 min for unilateral and 46.1 min for bilateral inguinal hernia repair [30]. The average follow-up was 30.4 months for PIRS and 24.4 months for open surgery (Table 1).

## Conclusions

The PIRS method can be considered a safe and feasible method for pediatric inguinal hernia repair. To perform the PIRS procedure, the surgeon only needs to gain basic laparoscopy experience. In the PIRS technique, the aim is to complete the procedure without mechanical trauma to the spermatic cord. With similar rates of complications and recurrence from the open surgical technique, PIRS has the potential of becoming a standard procedure for pediatric inguinal hernia repair. Randomized controlled trials with longer follow-ups are needed to compare the results with regard to fertility rates related to inguinal region surgery.

The laparoscopic percutaneous internal ring suturing technique is an alternative to open repair of indirect inguinal hernias in children. This technique requires only basic laparoscopy experience and has the advantages of preventing spermatic cord injury in males and excellent cosmetic outcomes in terms of surgical scars on the skin.
